# Investigation of Thermo-Hydraulics in a Lid-Driven Square Cavity with a Heated Hemispherical Obstacle at the Bottom

**DOI:** 10.3390/e26050408

**Published:** 2024-05-08

**Authors:** Farhan Lafta Rashid, Abbas Fadhil Khalaf, Arman Ameen, Mudhar A. Al-Obaidi

**Affiliations:** 1 Petroleum Engineering Department, College of Engineering, University of Kerbala, Karbala 56001, Iraq; farhan.lefta@uokerbala.edu.iq (F.L.R.); abbas.fadhil@uokerbala.edu.iq (A.F.K.); 2 Department of Building Engineering, Energy Systems and Sustainability Science, University of Gävle, 801 76 Gävle, Sweden; 3 Technical Institute of Baquba, Middle Technical University, Baquba 32001, Iraq; dr.mudhar.alaubedy@mtu.edu.iq; 4 Technical Instructor Training Institute, Middle Technical University, Baghdad 10074, Iraq

**Keywords:** square cavity, lid-driven cavity (LDC), moving or stationary walls, separation, wall attachment

## Abstract

Lid-driven cavity (LDC) flow is a significant area of study in fluid mechanics due to its common occurrence in engineering challenges. However, using numerical simulations (ANSYS Fluent) to accurately predict fluid flow and mixed convective heat transfer features, incorporating both a moving top wall and a heated hemispherical obstruction at the bottom, has not yet been attempted. This study aims to numerically demonstrate forced convection in a lid-driven square cavity (LDSC) with a moving top wall and a heated hemispherical obstacle at the bottom. The cavity is filled with a Newtonian fluid and subjected to a specific set of velocities (5, 10, 15, and 20 m/s) at the moving wall. The finite volume method is used to solve the governing equations using the Boussinesq approximation and the parallel flow assumption. The impact of various cavity geometries, as well as the influence of the moving top wall on fluid flow and heat transfer within the cavity, are evaluated. The results of this study indicate that the movement of the wall significantly disrupts the flow field inside the cavity, promoting excellent mixing between the flow field below the moving wall and within the cavity. The static pressure exhibits fluctuations, with the highest value observed at the top of the cavity of 1 m width (adjacent to the moving wall) and the lowest at 0.6 m. Furthermore, dynamic pressure experiences a linear increase until reaching its peak at 0.7 m, followed by a steady decrease toward the moving wall. The velocity of the internal surface fluctuates unpredictably along its length while other parameters remain relatively stable.

## 1. Introduction

Lid-driven cavity flow is an important field of study in fluid mechanics due to its common occurrence in engineering challenges, such as short-dwell and flexible blade coaters. Because of its simple shape and intricate flow patterns, this type of flow configuration can serve as a preliminary challenge for many numerical algorithms. Several relevant research studies have focused on this subject and its implications for engineering in recent years. Fluid flow with solid particles plays a significant role in a wide range of engineering applications, including coal combustion and particle separation [1,2]. The lid-driven (LD) flow in a cubical cavity has indeed attracted the attention of researchers because of its wide range of applications, which include electronic card cooling, food processing, crystal formation, and multi-screen devices used in nuclear reactors. Various types of restricted flow settings, including two- and three-dimensional cavity flows, are recognized issues in fluid dynamics that exhibit the main elements necessary for the transition to periodic and turbulent flows. Therefore, one of the primary challenges in understanding fluid dynamics is the flow structure inside cubical enclosures due to the cylindrical shape at the center [3,4]. Research in this field has shown significant interest in thoroughly examining physical perceptions as well as flow behavior in LD enclosures. Enclosures of different shapes (such as square, circular, and triangular) have been utilized to control or separate fluid flow [5]. Due to the LD cavity’s capability to evaluate the accuracy and efficiency of newly developed numerical methods, as well as its intrinsic desirability, numerous research studies have been reported in the literature. The initial numerical study by Burgraff [6] focused on two-dimensional lid-driven cavities. Ghia et al. [7] utilized a stream function–vorticity method combined with an implicit multigrid method to achieve accurate calculations in such contexts. Cortes et al. [8] investigated rectangular cavities with variable aspect ratios, expanding upon previous studies that were limited to square cavities with uniform aspect ratios. Bhopalam et al. [9] employed the lattice Boltzmann technique to estimate flows in double-sided cross-shaped LD cavities. This cavity configuration, with anti-parallel uniform wall motion, denoted as a complex geometry, was determined to be a symmetrized variation in the staggered LDC. The investigation of Reynolds numbers (Re) and oscillation frequencies for both uniform and oscillatory wall motions documented the motion and formation of primary and secondary vortices efficiently. To simulate the turbulence occurring inside a cubic LDC at Re 10,000, Samantaray et al. [10] combined large eddy simulation with the dynamic Smagorinsky model. Comparison with the joint probability distribution function revealed the presence of more consistent structures near the downstream wall, as indicated by quadrant analysis. Using the lattice Boltzmann technique, Hu [11] investigated the motion of a neutrally buoyant elliptical particle in a square cavity driven by the lid. The existence of a limit cycle, caused by the inertia of the elliptical particle, confines the motion within the boundaries of the square cavity. In this context, the vortex has been identified as the most prominent aspect of the elliptical particle’s motion in the square cavity. The boundary confinement tightens with increasing particle size, bringing the limit cycle closer to the center of the square cavity.

Samantaray and Das [12] employed a dynamic Smagorinsky model (DSM) to conduct intensive eddy simulations of incompressible flow inside a cubic lid-driven cavity (LDC) across a range of Re (1000, 3200, 5000, 10,000, and 15,000). The researchers found that even at a low Re of 3200, turbulence manifests as non-homogeneous and asymmetrical, with non-homogeneity worsening as Re increases. Specifically, turbulence in the core area of the cavity becomes more isotropic with increasing Re. Roy et al. [13] conducted multiple simulations and parametric investigations to determine the influence of material properties, obstacle geometry, and adhesion force on deposition patterns. They concluded that particles are frequently trapped in biofilm and areas near cavities with low Okubo–Weiss numbers, where relative vorticity predominates over local strains.

Golkarfard et al. [14] numerically investigated aerosol particle deposition in laminar mixed-convection flow using a lid-driven (LD) hollow with two heated barriers. It was also assumed that 2000 particles scattered uniformly in the flow regime and monitored using Lagrangian particle transport simulations had no effect on the fluid. Numerical simulations revealed that free convection significantly contributes to deposition. The main finding was that an increase in the Richardson number (Ri) leads to a decrease in deposition. Khan et al. [15] investigated the mixed convection flow of a hybrid nanofluid consisting of Al_2_O_3_CuH_2_O inside a trapezoidal cavity driven by a split lid. Within the cavity, a triangular-shaped cooling barrier was positioned. The horizontal base of the cavity was cooled, while the side walls were maintained adiabatic. A numerical solution using the Galerkin finite element approach coupled with physical boundary constraints was used. The findings of this investigation provided qualitative insights for enhancing cooling mechanisms for various thermal and electrical devices.

In another study, Khan et al. [16] presented a comprehensive computational investigation of Casson fluid hydromagnetic flow and heat transfer in a partially heated enclosed triangular cavity. Within the cavity, a cylindrical obstruction with various thermal boundary conditions was introduced. The finite element method (FEM) was utilized to solve the governing equations and examine the impact of different physical parameters on streamlines, isotherms, and local Nusselt numbers (Nu), including the Hartmann number (Hn). It was discovered that heat transfer inside the cavity is primarily influenced by the length of the heating element.

Khan et al. [17] investigated double-diffusive natural convection in a right-angle trapezoidal cavity filled with porous media using numerical simulations. The top and bottom horizontal boundaries were impermeable, while the inclined sidewall was maintained at a low concentration and temperature. The vertical left sidewall was kept at a high concentration and subjected to a fixed heat flux. It was found that lower values of the Lewis number (Ln) increased heat transfer, whereas larger values decreased it. The buoyancy parameter was shown to decrease the dimensionless temperature of the cavity while increasing the dimensionless concentration due to its strong influence on buoyancy. In another study, Khan et al. [18] explored natural convective heat transfer in various industrial applications, aiming to optimize performance. They conducted numerical simulations to analyze fluid flow and heat transfer within the system, as well as to investigate entropy generation, which is critical according to the thermodynamic Gouy–Stodola theorem. Utilizing non-dimensional primitive variables, they employed the Oberbeck–Galerkin finite element method to solve the Boussinesq equations. The researchers observed that the average Nu and Bejan numbers (Bn), as well as the average entropy generation, increased with the amplitude of the sinusoidal wall temperature. Additionally, increasing the non-uniform wall temperature wave number reduced the strength of energy transfer and Bn. An entropy assessment for triple-diffusive flow was conducted by Khan et al. [19]. They introduced a detailed model to analyze entropy production resulting from the variable chemical potential in NaCl and sucrose salts, considering heat and mass transfer, fluid friction, and porous medium effects. Both salt concentrations were found to be higher at the surface. The Boussinesq approximation and Darcy’s law were utilized to solve the dimensionless governing equations. Various combinations of variable concentration, velocity, and temperature were employed in the entropy generation model. This research revealed that aiding flows generate more entropy compared to opposing flows.

This study aims to establish a standard for an LD square cavity problem with a moving top wall and a bottom hemispherical barrier, aiming to characterize the underlying mechanics of the problem. This research aims to predict fluid flow and mixed convective heat transfer features while considering both a moving top wall and a heated hemispherical obstruction at the bottom. Specifically, the impact of various cavity geometries, as well as the influence of the moving top wall on fluid flow and heat transfer within the cavity, are addressed. To systematically conduct this aim and predict fluid flow and mixed convective heat transfer features, the fundamental mass, momentum, and energy equations presented in the open literature are used to express the steady state, incompressible, and two-dimensional laminar fluid flow. The finite volume method is then utilized to solve the governing equations using the Boussinesq approximation. 

There are several reasons behind the importance of accurately predicting the fluid flow and mixed convective heat transfer features, incorporating both a moving top wall and a heated hemispherical obstruction at the bottom. On top of this, perceiving the complicated interplay between forced convection due to the moving top wall and natural convection due to the heated hemispherical obstruction is essential for designing and optimizing the heat transfer processes in various applications besides achieving the desired performance. Heat exchangers, electronic cooling systems, and solar collectors are some examples of the applications. To the authors’ knowledge, this research represents a common problem in the relevant literature, and further investigation is needed into this topic.

## 2. Problem Description and Mathematical Modeling

**Figure 1:**
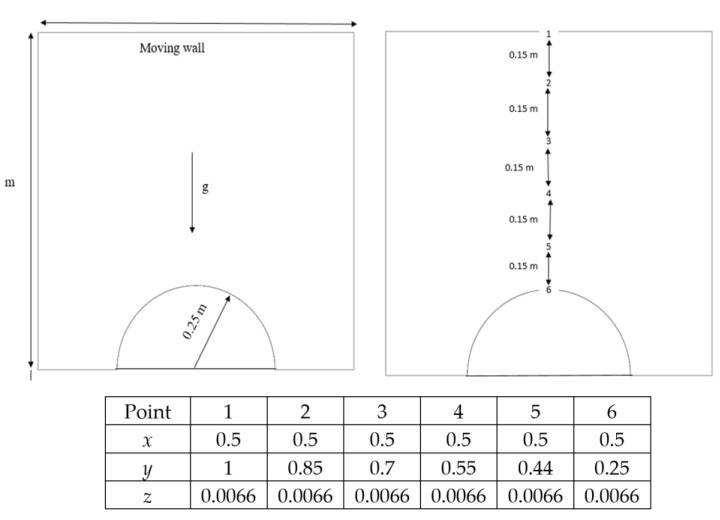
Dimensions and coordinates used in this study.

Figure 1 depicts fluid flow within a two-dimensional square cavity with a width of 1 m and a continuously moving top (lid) surface. The lid moves from left to right at a variable set of velocities: 5, 10, 15, and 20 m/s. The two vertical walls and the bottom of the cavity are perfectly insulated, maintaining a steady temperature of 25 °C for the lid surface. Additionally, a hemispherical barrier is fixed to the bottom wall at a steady temperature of 25 °C, with a radius of 0.25 m. The boundary conditions are established based on the following factors [11,20,21,22]:

1. The axis of symmetry.
2. The rotating periodicity (usually denoted as the sides of the wedge).
3. Inlet:

(1) $$u=\left(U,0,0\right),\nabla p.n=0,\tau =0$$

4. Outlet:

(2) $$\nabla u_{i}\cdot n=0,p=0,\nabla \tau_{ij}\cdot n=0$$

The fundamental relationships for incompressible, stable, and 2-dimensional flow are the mass, momentum, and energy as stated below [23,24,25,26]:

(3) $$\frac{\partial u}{\partial x}+\frac{\partial v}{\partial y}=0$$

(4) $$u\frac{\partial u}{\partial x}+v\frac{\partial u}{\partial y}=-\frac{1}{\rho }\frac{\partial P}{\partial x}+\frac{\mu }{\rho }\left(\frac{\partial^{2}u}{{\partial x}^{2}}+\frac{\partial^{2}u}{\partial y^{2}}\right)$$

(5) $$u\frac{\partial v}{\partial x}+v\frac{\partial v}{\partial y}=-\frac{1}{\rho }\frac{\partial P}{\partial y}+\frac{\mu }{\rho }\left(\frac{\partial^{2}v}{{\partial x}^{2}}+\frac{\partial^{2}v}{\partial y^{2}}\right)+g\beta (T-T_{C})$$

(6) $$\frac{\partial (\rho uC_{P}T)}{\partial x}+\frac{\partial (\rho vC_{P}T)}{\partial y}=k\left(\frac{\partial^{2}T}{{\partial x}^{2}}+\frac{\partial^{2}T}{\partial y^{2}}\right)$$

(7) $$\beta =\frac{1}{T_{f}}$$

u and v are the *x*- and *y*-components of velocity, respectively; P is the pressure; ρ and μ are the fluid density and dynamic viscosity, respectively; β is the coefficient of the thermal expansion; $T_{f}$ and T are the reference fluid temperature and fluid temperature, respectively; and k and g are the fluid thermal conductivity and the gravitational acceleration vector, respectively. 

The assumptions for mixed convection in an LDC with a heated hemispherical cavity at the bottom are as follows:

- The flow of air is considered two-dimensional steady, incompressible, and laminar flow.
- Internal heat generation is ignored.
- Radiation of heat transfer is considered negligible.
- The thermo-physical features are fixed. However, the density in the term of the body force in the momentum equation is treated following the Boussinesq approximation, giving rise to buoyancy forces.

Referring to the above presentation, it is important to ascertain that this study focuses on steady-state conditions. This means that the fluid flow and heat transfer features are assumed to be constant over time. The governing equations are, therefore, time-dependent, and the initial and boundary conditions are set to specify a steady-state condition. Considering the impact of forced convection on fluid flow and heat transfer, it is essential to clarify that the impact will be similar in both steady-state and unsteady-state conditions. However, the key difference is that in unsteady-state conditions, the flow and temperature fields will progress over time until they reach a steady state.

## 3. Simulation and the Numerical Test

Simulation and numerical testing offer numerous advantages over physical testing. These include reduced cost and time and increased accuracy, safety, and flexibility. These advantages make simulation and numerical testing important tools for engineers and designers, allowing them to test a wider range of design configurations more swiftly and inexpensively while also refining the safety and accuracy of the testing process [27,28]. Before conducting the numerical simulation in this study, a series of tasks must be completed and checked. The following are the two primary components used in the numerical simulation:

1. Developing the grids and examining the density of their constituent pieces are two steps toward reducing the amount of inaccuracy in the numerical findings.
2. Checking the accuracy of the numerical model that was employed.

**Figure 2:**
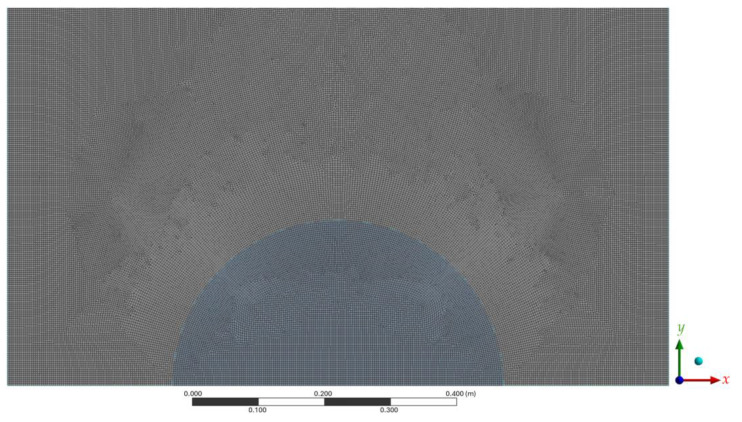
Mesh generation layout.

The grid of the examined space is made by using Gambit. The grid configuration is presented in Figure 2. The ratio value was used in each instance to determine the density of the components. The findings of this examination are shown in Table 1. It can be concluded that the number of elements in instance 2 is sufficient to deliver the desired level of satisfaction. This particular stage is referred to as the grid independence test. 

The numerical code in ANSYS Fluent was employed to address the problem posed by this investigation. The code initializes the initial conditions (Equations (1) and (2)) before transforming the differential equations into a matrix system using the finite volume approach. A high-resolution technique was applied to solve the convective terms of the matrix system. The SIMPLEC method was employed to couple pressure and velocity. Calculated results were considered acceptable, with errors of 10^−6^ for energy equations and 10^−8^ for momentum equations, respectively.

### Model Validation

**Figure 3:**
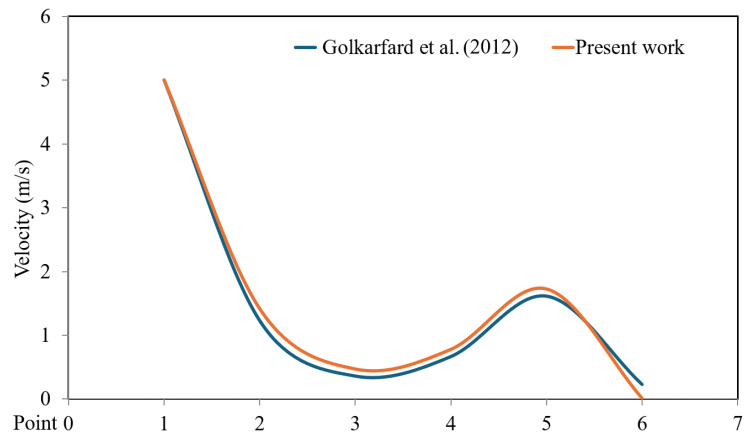
Comparison of velocity distributions between the results of this study and Golkarfard et al. [14] for a top moving wall velocity of 5 m/s.

Golkarfard et al. [14] studied aerosol particle deposition in a lid-driven hollow with two heated barriers. They introduced the LD square cavity with a hemispherical obstacle. The LD configuration features a top moving wall velocity of 5 m/s. This study addresses the issue presented by Golkarfard et al. [14] using the numerical method described herein. Figure 3 presents the velocity distribution findings for a top moving wall velocity of 5 m/s from both Golkarfard et al. [14] and this study. This comparison clearly demonstrates the accuracy of the present method. 

Despite the acceptable accuracy of utilizing the finite element approach to solve the governing equations, which addresses the velocity distribution for 5 m/s of top moving wall velocity (Figure 3), one can expect that the utilization of fourth-order finite difference numerical schemes for the incompressible fluid with Boussinesq approximation would be of higher accuracy to precisely represent the velocity distribution besides demonstrating actual fluid flow and temperature fields for the case of the forced convection in the lid-driven square cavity (LDSC) with a heated hemispherical obstacle.

## 4. Results and Discussion

### 4.1. Velocity, Pressure, and Temperature Distributions at Power Densities of 100 and 200 W/m^3^

This section presents the influences of the moving wall on pressure, velocity, and temperature distribution contours in the square cavity while utilizing different power densities of 100 and 200 W/m^3^ of the heated hemispherical obstacle at the bottom (demonstrated as a heat source). It is evident that the movement of the wall significantly disrupts the flow field inside the cavity, leading to excellent mixing between the flow field below the moving wall and within the cavity. Physically, forced convection arises from two mechanisms: airflow entering the cavity and the additional shear force caused by the movement of the wall. Additionally, it is observed that the streamlines are highly influenced by the direction of the wall movement, clustering near the moving wall due to the shear force generated. The movement of the wall also eliminates the stationary region at the lower corners of the side wall of the cavity, facilitating the extension of vortices further inside the cavity. 

**Figure 4:**
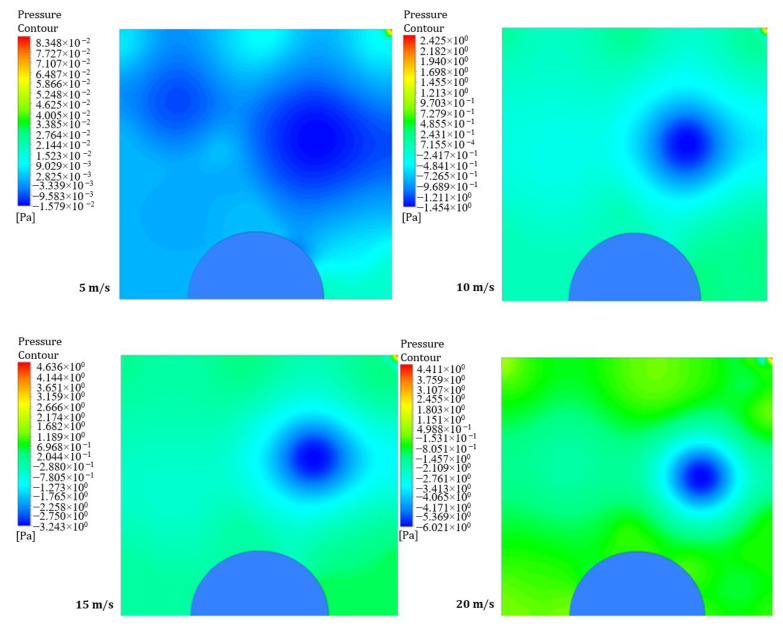
Contours of pressure distribution for different moving wall velocities at a power density of 100 W/m^3^.

**Figure 5:**
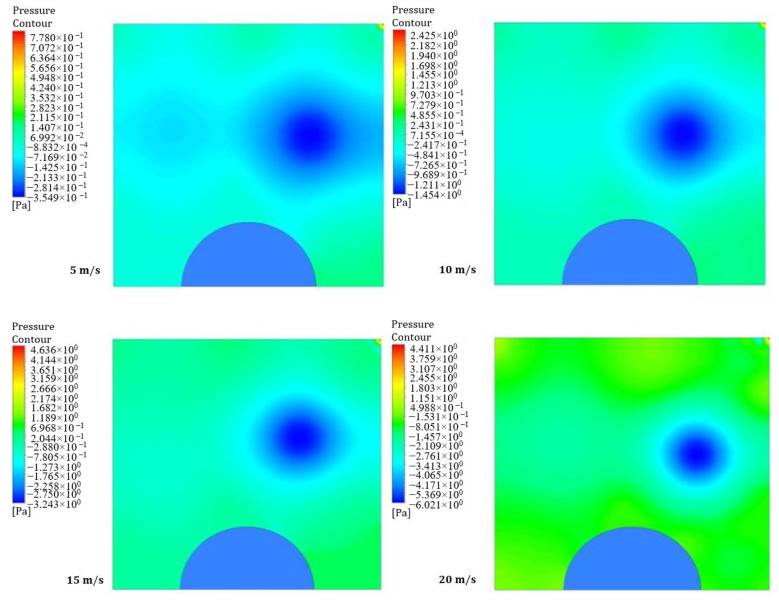
Contours of pressure distribution for different moving wall velocities at a power density of 200 W/m^3^.

Static pressure refers to the pressure on the surface of a uniformly moving wall, while dynamic pressure is experienced when a surface moves in the fluid’s direction. Figure 4 and Figure 5 illustrate four contours of pressure distribution for different moving wall velocities (5, 10, 15, and 20 m/s) at power densities of 100 and 200 W/m^3^, respectively. The figures show that an increase in the moving wall velocity leads to noticeable differences in the flow and thermal fields inside the LD square cavity. The increase in power density enhances natural convection generated from the heated obstacle, increasing the mixing between hot air surrounding the hemispherical obstacle and cold air entering the square cavity under the action of the moving top wall, consequently increasing the pressure inside the cavity.

**Figure 6:**
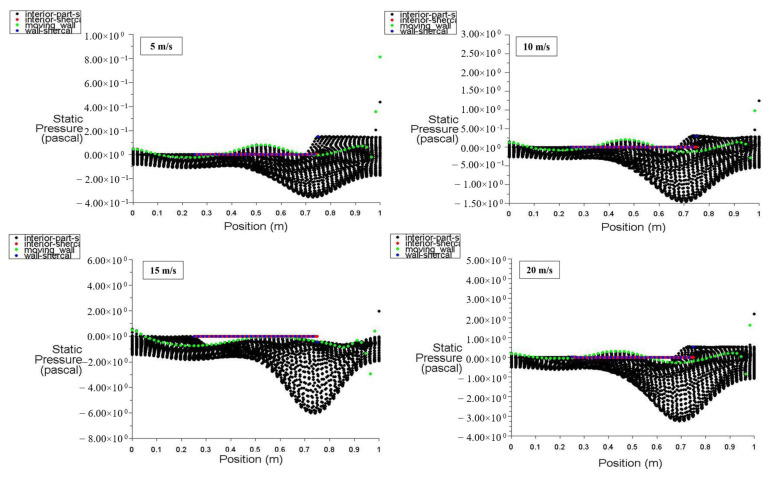
Static pressure distribution versus position at a power density of 100 W/m^3^ for different moving wall velocities.

Figure 6 illustrates the distribution of static pressure along the positions of the moving wall, bottom wall, left wall, right wall, and interior surface at a power density of 100 W/m^3^. It is evident that there is fluctuation in the static pressure values for different moving wall velocities of 5, 10, 15, and 20 m/s, starting from zero at the bottom of the cavity, with the lowest value occurring between 0.7 and 0.75 m and the highest value at 1 m, which is in contact with the moving wall. At point 1 (top wall location), the static pressure starts at a negative (vacuum) value and maximizes at 2.5 Pa due to the effect of the top moving wall on the air near the right wall, forcing it to escape from the cavity. The interior surface exhibits random static pressure variations along its position due to the recirculation of air entering and exiting the cavity.

**Figure 7:**
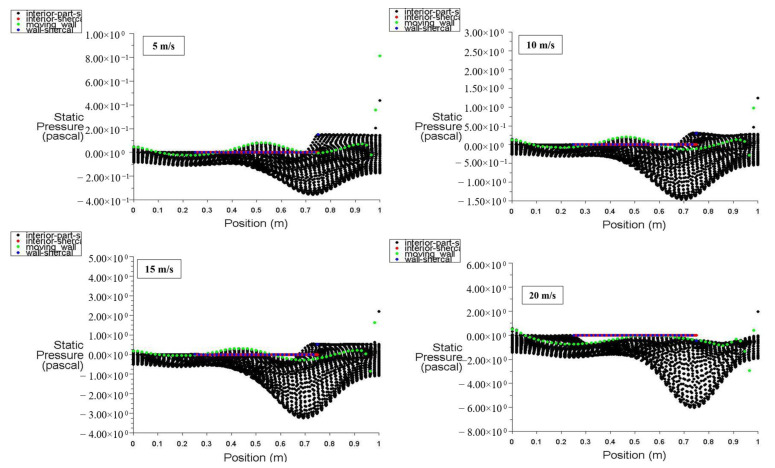
Static pressure distribution versus position at a power density of 200 W/m^3^ for different moving wall velocities.

With a power density of 200 W/m^3^, Figure 7 depicts the static pressure distribution for the moving wall, bottom wall, left wall, right wall, and inner surface positions. The heated hemispherical obstacle located at the center-bottom of the cavity produces more natural convection as its power density increases, enhancing the mixing of hot air rising from the heated obstacle with cold air influenced by the moving top wall, thus increasing the static pressure inside the cavity.

**Figure 8:**
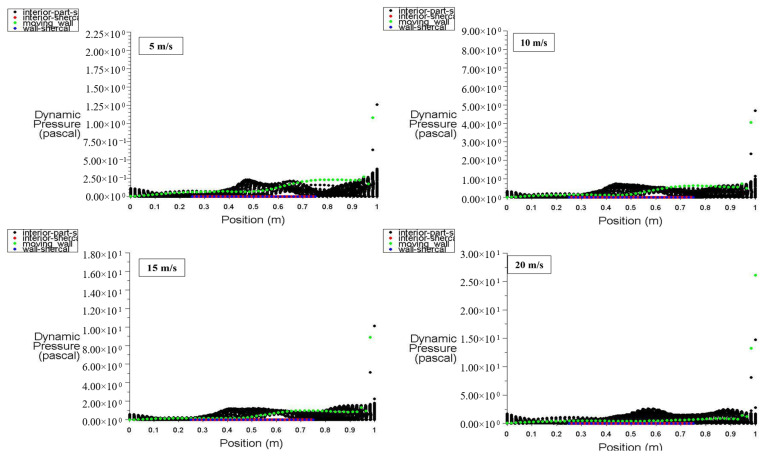
Dynamic pressure distribution versus position at a power density of 100 W/m^3^ for different moving wall velocities.

Figure 8 illustrates the distribution of static pressure along the positions of the moving wall, bottom wall, left wall, right wall, and interior surface at a power density of 100 W/m^3^. It can be observed that the dynamic pressure for the moving wall is at its lowest value at the bottom of the cavity, increases to 1.9 Pa (maximum) at 0.7 m, and then increases to 9 Pa at the top of the zone (at 1 m) using 15 m/s of moving wall velocity. At point 1 (top wall location), the dynamic pressure starts at zero and hits 10 Pa due to the effect of the top moving wall on the air near the right wall, forcing it to escape from the cavity. The interior surface exhibits random dynamic pressure variations along its position due to the fluctuation of the air stream entering and leaving the cavity under the influence of the top moving wall.

**Figure 9:**
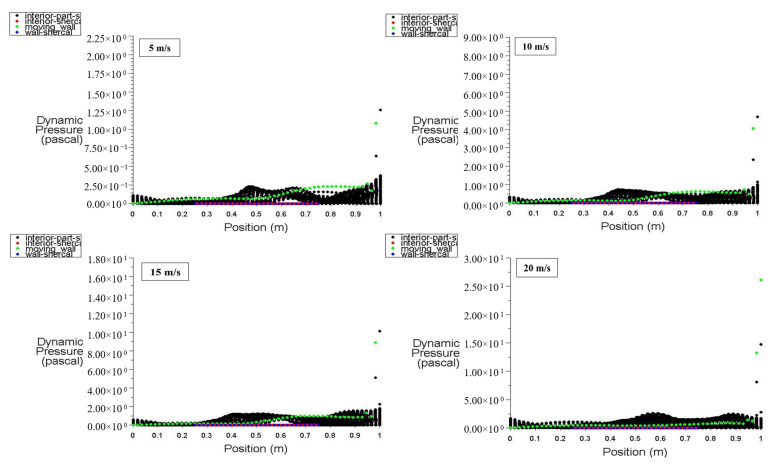
Dynamic pressure distribution versus position at a power density of 200 W/m^3^ for different moving wall velocities.

With a power density of 200 W/m^3^, Figure 9 shows that an increase in the power density of the heated hemispherical obstacle leads to an increase in natural convection due to the heating effect. Consequently, the increased mixing between hot air rising from the obstacle and cold air entering the cavity results in an increase in dynamic pressure inside the cavity.

**Figure 10:**
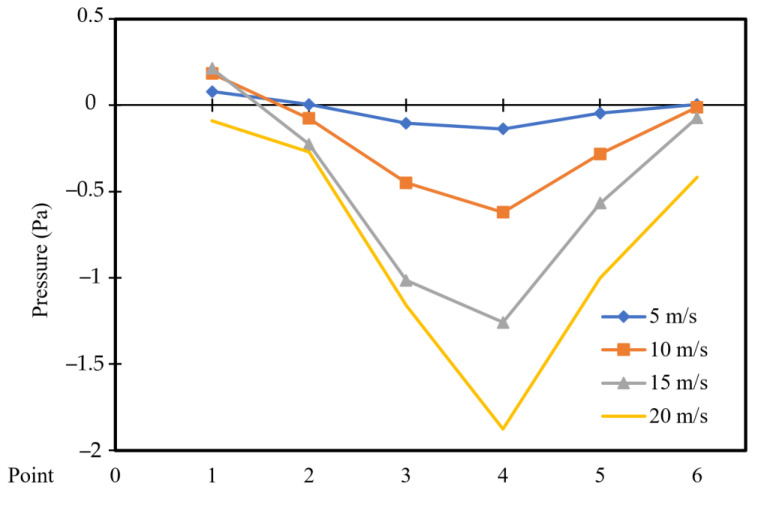
Pressure distribution versus position at a power density of 100 W/m^3^ for different moving wall velocities.

**Figure 11:**
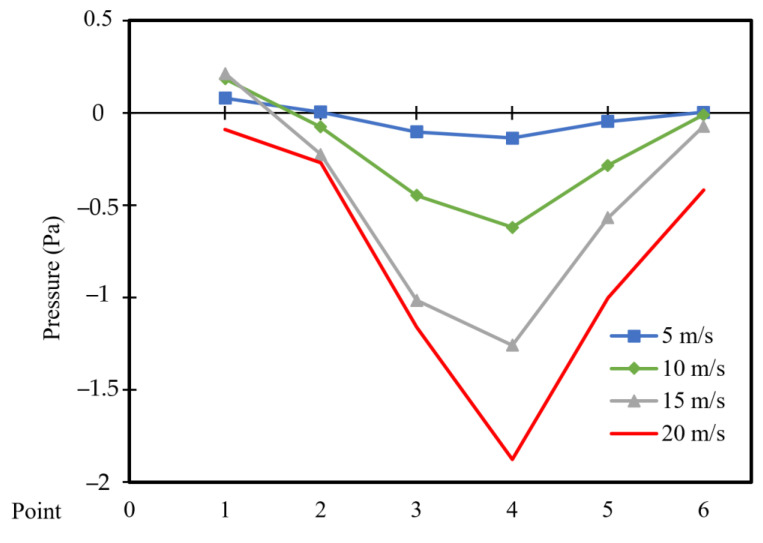
Pressure distribution versus position at a power density of 200 W/m^3^ for different moving wall velocities.

Figure 10 and Figure 11 depict the pressure distribution across six points located vertically in the cavity for different moving wall velocities with power densities of 100 W/m^3^ and 200 W/m^3^, respectively. 

A symmetric pressure distribution can be observed along the square cavity, with the highest value at point 1 and the lowest value at point 4 (vacuum). Point 6 exhibits lower pressure than point 1, as the movement of the wall influences the air near the right wall, causing it to escape from the cavity and reducing the pressure at point 6. Additionally, an increase in moving wall velocity results in a decrease in pressure (vacuum) distribution throughout the cavity (Figure 10). However, Figure 11 indicates that an increase in power density has no effect on the pressure distribution across the six points.

**Figure 12:**
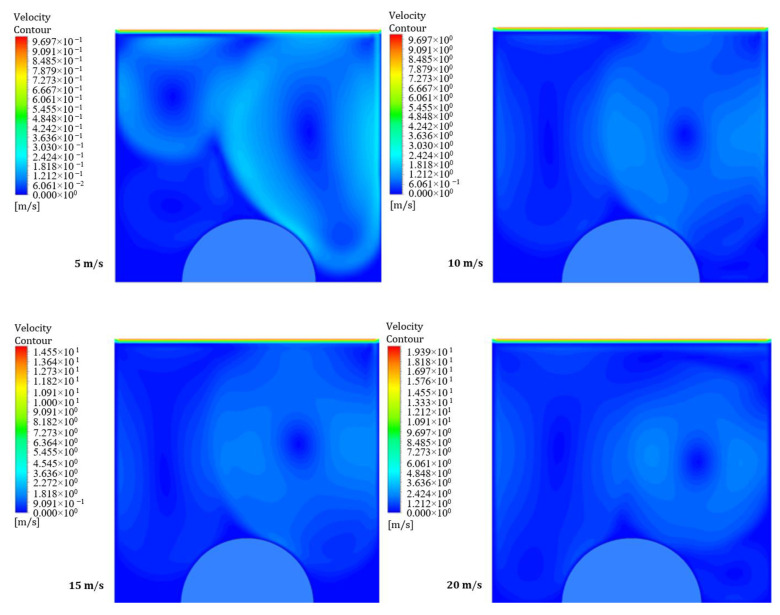
Contours of velocity distribution for different moving wall velocities at a power density of 100 W/m^3^.

**Figure 13:**
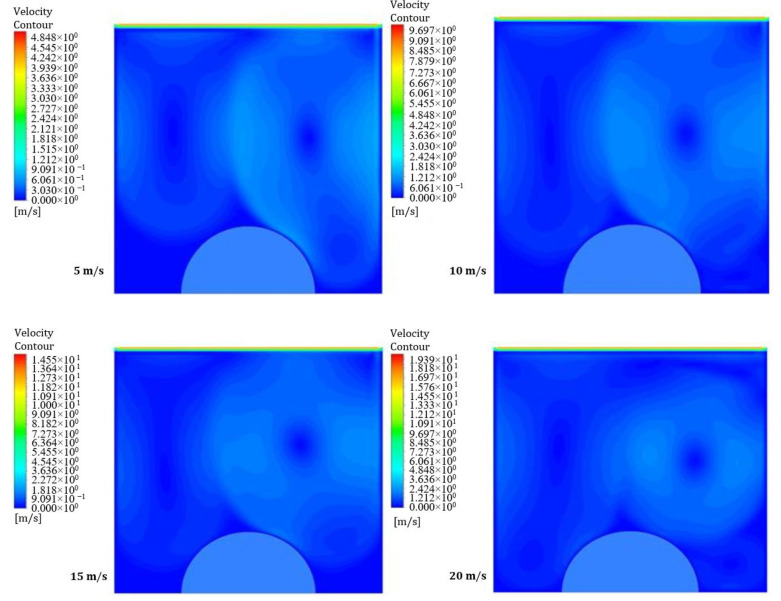
Contours of velocity distribution for different moving wall velocities at a power density of 200 W/m^3^.

Figure 12 and Figure 13 illustrate the influence of a moving wall on the velocity distribution patterns in the square hollow for power densities of 100 and 200 W/m^3^, respectively. These figures show four velocity distribution patterns with different moving wall velocities of 5, 10, 15, and 20 m/s. The flow field within the cavity is noticeably disrupted by the movement of the wall. Moreover, these findings suggest that a moving wall promotes effective blending between the cavity and the underlying flow field. Additionally, Figure 13 shows that an increase in power density has a minimal effect on the velocity distribution within the square cavity.

**Figure 14:**
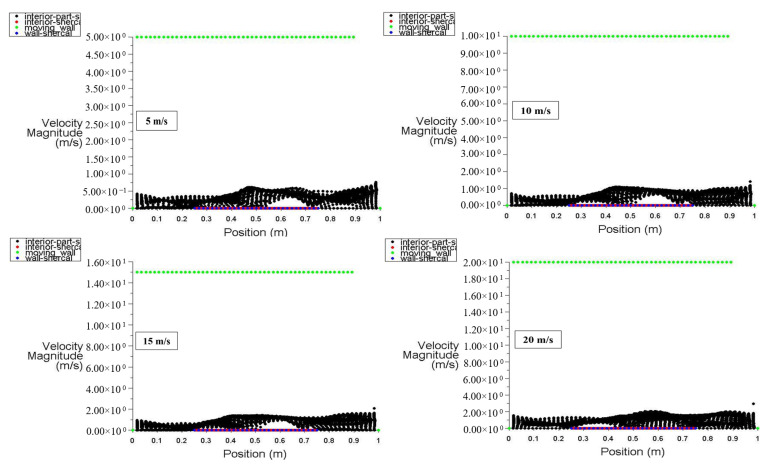
Velocity distribution versus position for different moving wall velocities at a power density of 100 W/m^3^.

**Figure 15:**
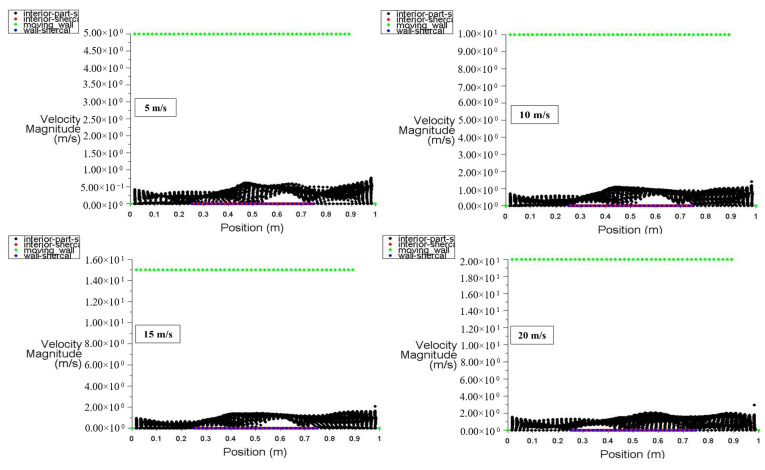
Velocity distribution versus position for different moving wall velocities at a power density of 200 W/m^3^.

At power densities of 100 and 200 W/m^3^, respectively, Figure 14 and Figure 15 depict the velocity distribution along the locations of the moving top wall, bottom wall, interior surface, left wall, and right wall. These figures specifically show the velocity distribution for power densities of 100 and 200 W/m^3^, with the moving walls set at specific velocities of 5, 10, 15, and 20 m/s. Figure 14 illustrates that the velocity of the interior surface fluctuates significantly along its position while remaining static for the other surfaces. Additionally, Figure 15 clearly demonstrates that increasing power density has no discernible impact on the velocity distribution inside the square cavity.

**Figure 16:**
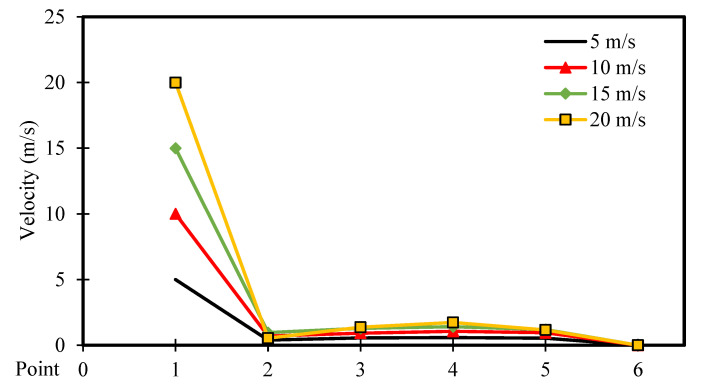
Airflow velocity distribution versus position for different moving wall velocities at a power density of 100 W/m^3^.

**Figure 17:**
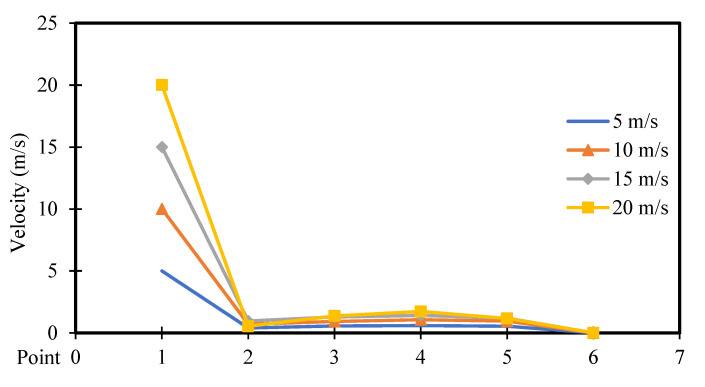
Airflow velocity distribution versus position for different moving wall velocities at a power density of 200 W/m^3^.

Figure 16 and Figure 17 depict the velocity distribution across six selected points along the square cavity for different moving wall velocities of 5, 10, 15, and 20 m/s, with power densities of 100 W/m^3^ and 200 W/m^3^, respectively. It can be observed that the velocity increases to reach its highest value at point 1, then decreases to the lowest value at point 2, and remains constant for the other points. Additionally, an increase in moving wall velocity results in a higher velocity distribution throughout the cavity. However, Figure 17 confirms that the increase in power density has a negligible influence on the velocity distribution across the points when using a power density of 200 W/m^3^.

**Figure 18:**
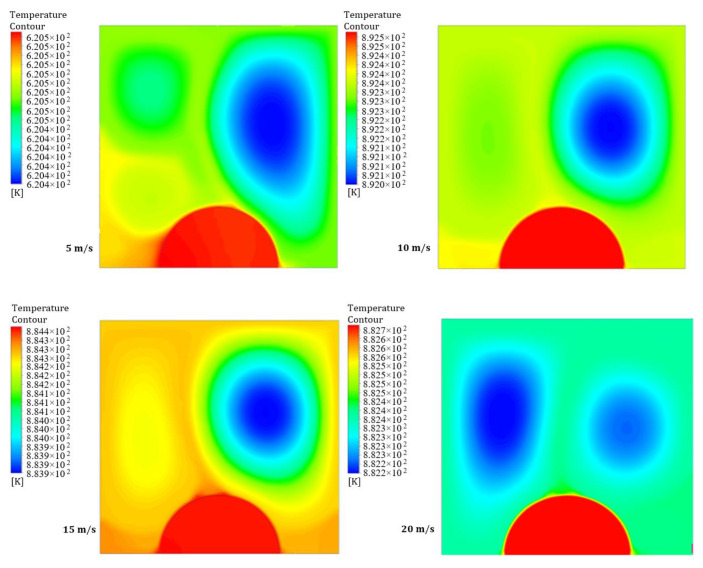
Contours of temperature distribution at a power density of 100 W/m^3^ for different moving wall velocities.

**Figure 19:**
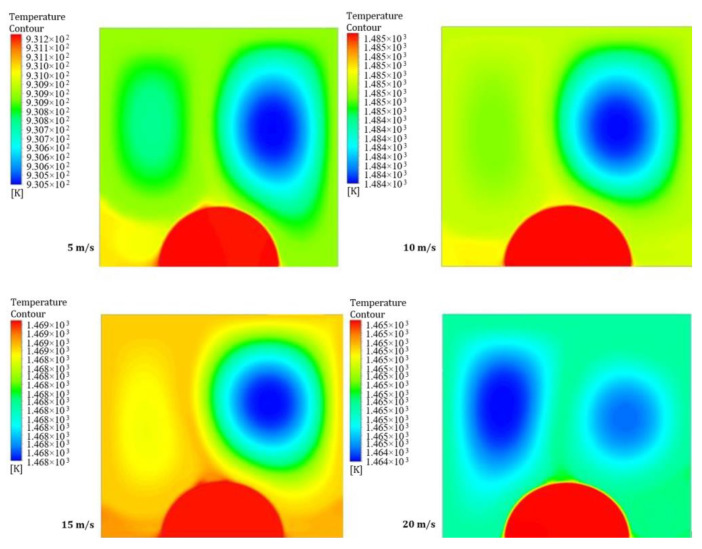
Contours of temperature distribution at a power density of 200 W/m^3^ for different moving wall velocities.

Figure 18 and Figure 19 illustrate four contours of temperature distribution for different moving wall velocities of 5, 10, 15, and 20 m/s at two power densities of 100 and 200 W/m^3^, respectively. These figures demonstrate clear differences in the flow and thermal fields inside the LD square cavity as the moving wall velocity increases. Forced convection arises from two factors: the airflow into the cavity and the additional shear stress caused by the moving wall. Moreover, the heated obstruction induces more natural convection due to the higher power density, leading to increased mixing of hot air rising from the heated obstacle and cold air entering the cavity, resulting in higher temperature distribution within the cavity.

**Figure 20:**
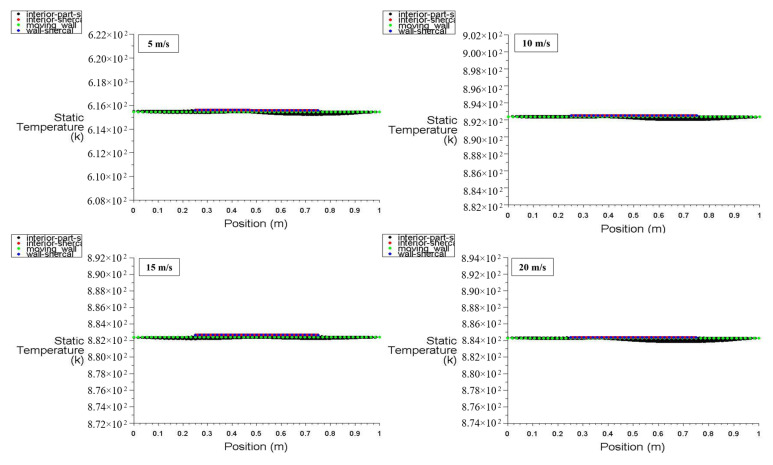
Static temperature distribution versus position for different moving wall velocities at a power density of 100 W/m^3^.

**Figure 21:**
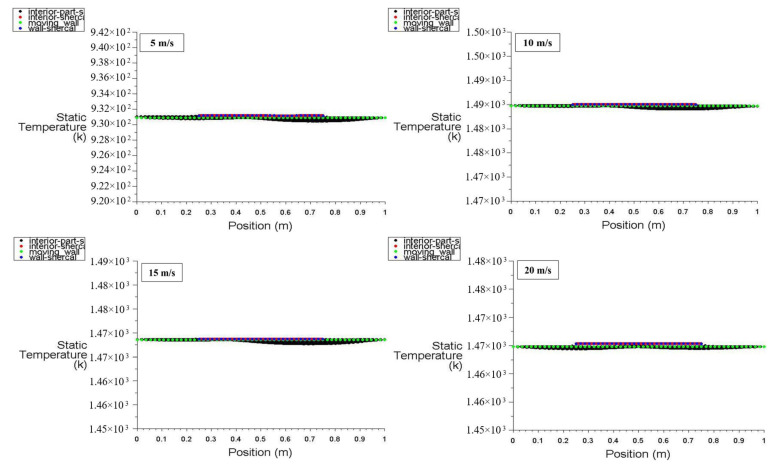
Static temperature distribution versus position for different moving wall velocities at a power density of 200 W/m^3^.

Figure 20 and Figure 21 display the static temperature distribution along the positions of the moving wall, bottom wall, interior surface, left wall, and right wall at power densities of 100 and 200 W/m^3^, respectively. These figures specifically illustrate the static temperature distribution for different moving wall velocities of 5, 10, 15, and 20 m/s. It is evident that the static temperature of the interior surface varies insignificantly along its position (Figure 20), while the temperatures of the other surfaces remain constant. Notably, Figure 21 demonstrates that an increase in power density of the heated hemispherical obstacle has a significant effect on the static temperature distribution inside the square cavity, with a clear increment.

**Figure 22:**
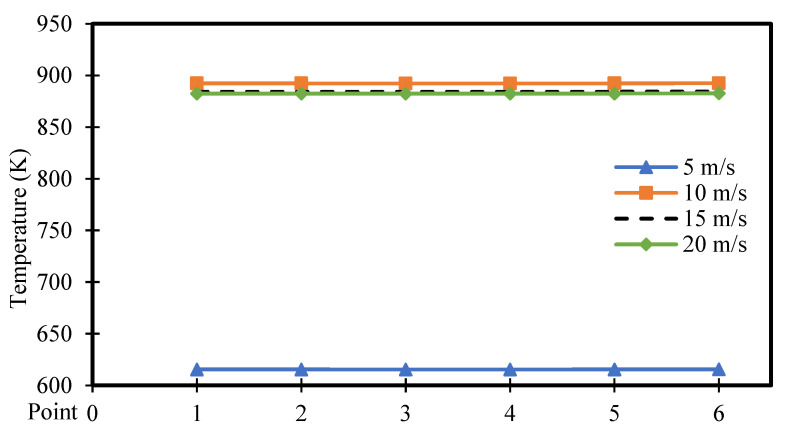
Temperature distribution versus position for different moving wall velocities at a power density of 100 W/m^3^.

**Figure 23:**
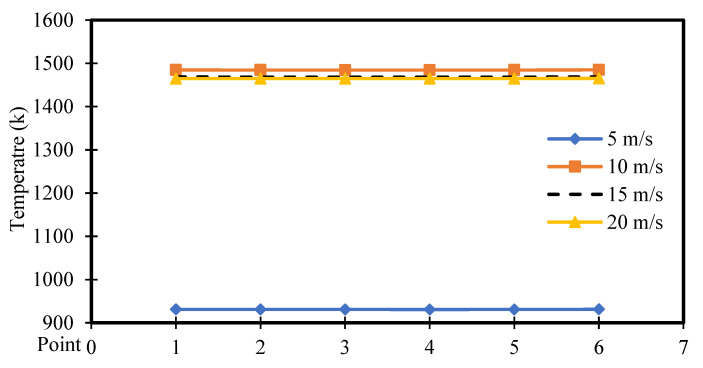
Temperature distribution versus position for different moving wall velocities at a power density of 200 W/m^3^.

Figure 22 and Figure 23 exhibit the temperature distribution across six points within the square cavity for power densities of 100 and 200 W/m^3^, respectively. Figure 22 illustrates that the temperature remains approximately constant along the points. These figures specifically display the temperature distribution for different moving wall velocities of 5, 10, 15, and 20 m/s. The increase in moving wall velocity enhances the mixing between the hot air rising from the heated hemispherical obstacle and the cold air coming from the moving top wall. Consequently, this increase in mixing results in an increased temperature distribution when the moving wall velocity varies from 5 to 10 m/s. However, moving wall velocities > 10 m/s led to a reduction in temperature, as the higher velocities cause more cold air to enter the cavity, thereby reducing the temperature. Moreover, the increase in power density amplifies the natural convection generated from the heated obstacle, leading to increased mixing between hot and cold air. Consequently, this action results in an increased temperature distribution inside the cavity, as seen in Figure 23.

**Figure 24:**
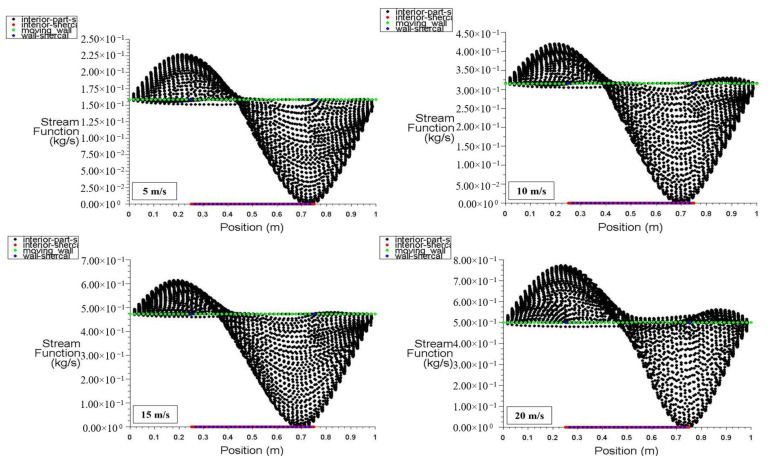
Stream function distribution versus position for different moving wall velocities at a power density of 100 W/m^3^.

**Figure 25:**
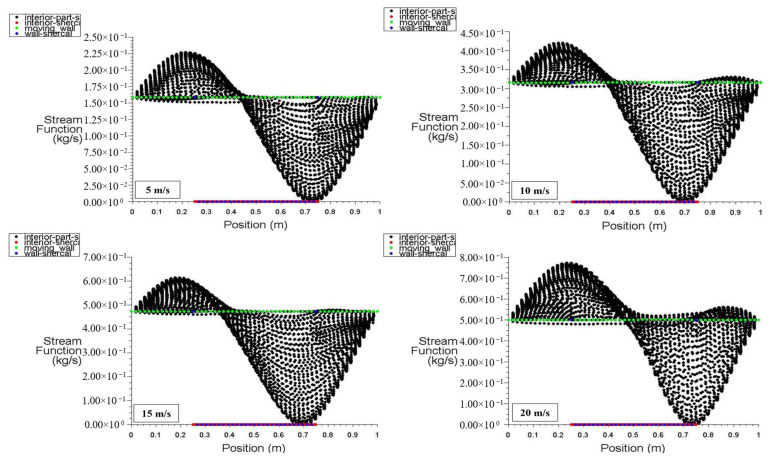
Stream function distribution versus position for different moving wall velocities at a power density of 200 W/m^3^.

Figure 24 and Figure 25 present the stream function distribution for the moving wall, bottom wall, interior surface, left wall, and right wall across the six points in the cavity for power densities of 100 and 200 W/m^3^, respectively. These figures specifically display the stream function distribution for different moving wall velocities of 5, 10, 15, and 20 m/s. It can be observed that the stream function of the interior surface follows a sinusoidal pattern and varies along the position. The lowest value occurs at 0.43 m (Figure 24), while the values for the other surfaces remain constant along the positions. Additionally, Figure 25 demonstrates that the increase in power density has no effect on the stream function distribution throughout the square cavity.

### 4.2. Comparison of Variable Moving Wall Velocities

**Figure 26:**
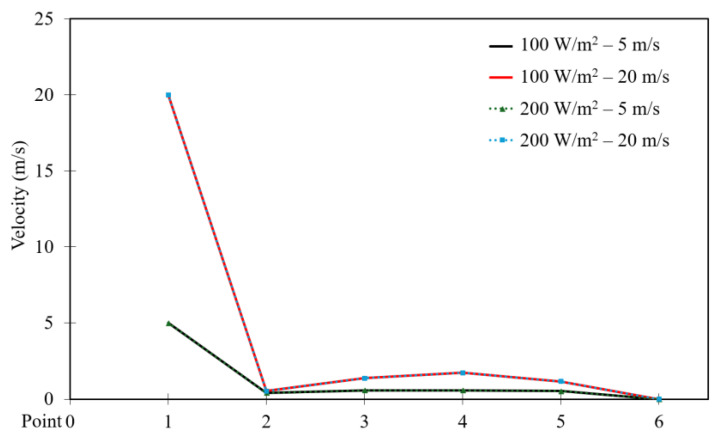
Comparison of velocity distribution versus point number for different moving wall velocities and power densities.

**Figure 27:**
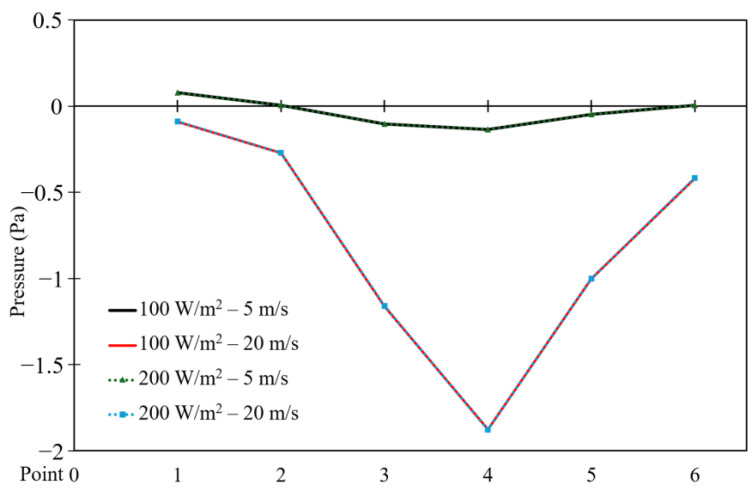
Comparison of pressure distribution versus point number for different moving wall velocities and power densities.

**Figure 28:**
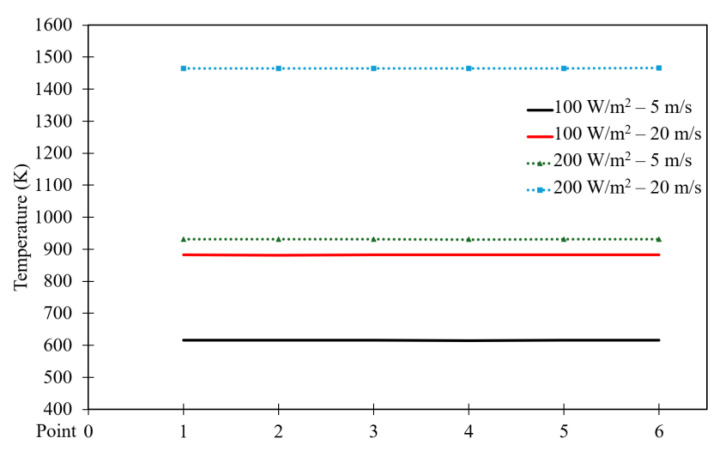
Comparison of temperature distribution versus point number for different moving wall velocities at different power densities.

This section compares the velocity, pressure, and temperature profiles considering variable top moving wall velocities (only 5 and 20 m/s) and power densities (100 and 200 W/m^3^). Figure 26, Figure 27 and Figure 28 present the numerical results of velocity, pressure, and temperature distribution, respectively, in response to the variations in top moving wall velocity and power densities. Figure 26 illustrates the distribution of air velocity along the position (the six points). It shows that the velocity peaks at point 1 and then decreases to its lowest value at point 2, remaining constant at the other points. The results indicate that increasing the moving wall velocity enhances airflow velocity while increasing power density has no influence on velocity distribution. Figure 27 displays the pressure distribution along the position (the six points). It demonstrates a symmetrical distribution along the two halves of the cavity, with the highest value at point 1, slightly lower at point 6, and the lowest at point 4. Increasing the moving wall velocity increases pressure distribution while increasing power density has no effect on pressure distribution.

Figure 28 shows the temperature distribution along the six points in the square cavity. It indicates that the temperature remains approximately constant across the points. Notably, increasing moving wall velocity reduces temperature distribution, while increasing power density increases it.

## 5. Conclusions

Fluid flow in an LDC is a classic benchmarking concern extensively explored by various researchers due to its simple geometric form and wide range of flow properties, including corner eddies, bifurcation, and transition to turbulence. This study aimed to replicate the mechanics underlying an LD square cavity using ANSYS Fluent to predict fluid flow and mixed convective heat transfer features involving both a moving top wall and a hemispherical heated barrier at the bottom. This work presented the outcomes of various combinations within the cavity with specific geometries, as well as an examination of the influence of the top moving wall on fluid flow and heat transfer in the cavity. The following conclusions were drawn:

1. The movement of the wall significantly disturbs the flow field inside the cavity, facilitating excellent mixing between the flow field below the moving wall and the cavity.
2. There is a fluctuation in static pressure, with the lowest value occurring between 0.7 and 0.75 m and the highest at the contact with the moving wall (1 m).
3. Dynamic pressure linearly increases until it reaches its peak at 0.7 m, then decreases linearly until point 1 (moving wall location).
4. Pressure is lowest at the bottom of the cavity, peaks at 0.7 m (1.9 Pa), and increases to 9 Pa at the top of the zone.
5. The velocity of the interior surface varies randomly along the position, while the velocities of the other surfaces remain constant.
6. Velocity peaks at point 1, decreases to its lowest at point 2, and remains constant through the other points.
7. Pressure is distributed symmetrically along the two halves of the cavity, with the highest value occurring at point 1, slightly less at point 6, and the lowest at point 4 (vacuum).

This study was conducted based on the assumption of a two-dimensional analysis of the fluid flow and heat transfer in the LDSC at steady-state conditions. Indeed, the unsteady state equations and time evolution would be interesting to investigate in future research to capture the transient behavior of the fluid flow and heat transfer. This case is quite necessary if the initial conditions or boundary conditions are time-dependent or if the fluid flow is turbulent. Furthermore, the investigation with a three-dimensional analysis is important to rectify if the fluid flow will be influenced by forces or boundary conditions that are not aligned with the plane of the cavity.

## Figures and Tables

**Table 1:** Grid independence test using a top moving wall velocity of 5 m/s.

Density	Case	Total Cell Number	Velocity (m/s)	Difference (%)
0.1	1	43,075	4.87	0.24
	2	100,550	4.90	0.09
	3	223,300	4.98	0.08
	4	321,500	5.00	0.08
0.2	1	42,020	4.76	0.48
	2	86,240	4.82	0.025
	3	174,660	4.87	0.023
	4	271,100	4.99	0.023
0.3	1	33,176	4.91	2.30
	2	70,552	4.93	0.22
	3	140,315	4.94	0.22
	4	221,000	4.92	0.22

## Data Availability

Data are contained within this article.

## Abbreviations

Symbol	Definition
Bn	Bejan number (-)
DSM	Dynamic Smagorinsky model
FEM	Finite element method
Hn	Hartmann number (-)
LD	Lid-driven
LDC	Lid-driven cavity
LDSC	Lid-driven square cavity
Ln	Lewis number (-)
Nu	Nusselt number (-)
Re	Reynolds number (-)
Ri	Richardson number (-)
